# Effects of undernutrition on survival of human immunodeficiency virus positive children on antiretroviral therapy

**DOI:** 10.1186/s13052-018-0472-2

**Published:** 2018-02-27

**Authors:** Animut Alebel, Fasil Wagnew, Cheru Tesema, Getiye Dejenu Kibret, Pammla Petrucka, Setegn Eshite

**Affiliations:** 1 College of Health Sciences, Debre Markos University, Debre Markos, Ethiopia; 2 College of Medicine and Health Sciences, University of Gondar, Gondar, Ethiopia; 3 College of Nursing, University of Saskatchewan, Saskatoon, Canada; 4 School of Life Sciences and Bioengineering, Nelson Mandela African Institute of Science and Technology, Arusha, Tanzania

**Keywords:** Anti-retroviral therapy (ART), Children, HIV, Mortality, Undernutrition

## Abstract

**Background:**

The relationship between undernutrition and HIV is bidirectional, ultimately contributing to quality of life and survival of affected individuals. Ethiopia is a sub-Saharan nation influenced by both undernutrition and HIV. In Ethiopia, although individuals are often dually impacted, the effect of undernutrition on the survival of HIV positive children on anti-retroviral therapy (ART) has not been well investigated. Therefore, this study assessed the effect of undernutrition on survival rates of HIV positive children on ART in Amhara Regional State of Ethiopia.

**Methods:**

An institution-based retrospective cohort study was conducted among 390 HIV positive children on ART from the 1st of January, 2012 to the 28th of February, 2017 in Amhara Regional State Referral Hospitals. A simple random sampling technique was used to select the study participants. Data were extracted by reviewing patients’ ART intake and follow-up forms. Data were entered into Epi-Data Version 3.1, and analysis was done using STATA Version 13. The Kaplan-Meier survival curve was used to estimate the cumulative survival time of the sample. Log rank tests were employed to compare the survival time between different categories of explanatory variables. Bivariable and multivariable Cox proportional hazards models were fitted to identify predictors of mortality.

**Results:**

Among the 390 records included in the final analysis, 9.7% of the individuals died within the follow-up period. In this study, the overall mortality rate was found to be 4.4 per 100 child-years (95% CI: 3.2, 6.0) while undernourished children had a lower survival time than well-nourished children. Low hemoglobin level (AHR: 3.2, 95% CI: 1.4, 7.4), CD4 cell count or percent below the threshold (AHR: 5.2, 95% CI: 1.9, 14.1), severe stunting (AHR: 3.9, 95% CI: 1.7, 9.4), severe wasting (AHR: 3.0, 95% CI: 1.3, 6.9) and advanced disease stage (III and IV) (AHR: 2.6, 95% CI: 1.1, 6.6) were found to be predictors of mortality.

**Conclusion:**

There was a high rate of mortality. A significant difference was observed in the survival rate of undernourished and well-nourished children. Low hemoglobin level, CD4 count or percent below the threshold, severe wasting, severe stunting, and advanced disease stage were found to be predictors of mortality.

## Background

The pandemic of Human Immunodeficiency Virus (HIV) creates an enormous challenge to the survival of humankind [1]. HIV develops very rapidly among infants and children, unless early initiation of ART, almost 33% of infants living with HIV die before their first birthday, and almost 50% die before the age of two years [2]. Comparable findings were also reported from Ethiopia, which supports this evidence [1, 3]. By the end of 2016, an estimated 2.1 million children under the age of 15 were living with HIV, of which approximately 64% are from sub-Saharan Africa [4]. Ethiopia is one of the sub-Saharan countries with the highest prevalence of HIV. The country has approximately 57,132, children (0-14 years) living with HIV and 1276 new infections are anticipated in 2017 [5].

Malnutrition refers to both undernutrition and overnutrition [6]; however, according to World Food Program definition, malnutrition refers to undernutrition unless otherwise specified [7]. Undernutrition is a major public health problem in countries with high prevalence of HIV. Independent of HIV, sub-Saharan Africa is the region of the world most seriously affected by malnutrition, reporting 21% of children under-five-year as underweight, 39% as stunted, and 9% as wasted [8]. Ethiopia is one of the sub-Saharan African nations largely affected by malnutrition and HIV. Malnutrition is attributed as the underlying cause in 57% of child mortality in Ethiopia. Despite significant progress in the reduction of child malnutrition, it remains widespread and a serious public health problem of Ethiopian children. As indicated by the 2016 Ethiopian Demographic and Health Survey report, 38%, 24% and 10% of under-five children were stunted, underweight and wasted respectively [9].

The relationship between malnutrition and HIV is bidirectional. Malnutrition may increase the progression of HIV infection to Acquired Immunodeficiency Syndrome (AIDS) stage by weakening the immune functions of the body. The interplay between HIV and malnutrition is cumulative with each negatively affecting the immune system [10–12] amongst people living with HIV/AIDS, resulting in malnutrition increasing the risk of morbidity and mortality, and potentially reducing the efficacy of anti-retroviral therapy (ART) [13]. Individuals, who were malnourished at initiation of ART, experience lower survival rates compared to well-nourished counterparts [14, 15]. Study findings indicated that even a relatively small departure in weight (5%) is associated with decreased survival rate [15].

Despite, the remarkable effects of Highly Active Antiretroviral Therapy (HAART) in the reduction of morbidity and mortality rate [1], all ART users may not equally responded to the therapy. In response to ART, there may be individual variability with some individuals having a lower progression in the recovery rate of immune function and remains at higher risk of dying from opportunistic infections. Therefore, it is better to consider another factor like nutritional status, which affecting immune response beside to ART provision [16].

In Ethiopia, different studies have been conducted to determine the predictors of mortality among HIV positive children on ART [1, 3, 17–19]. These studies showed that low hemoglobin counts, low CD4 counts below the threshold level, severe immune deficiency, age of the child at initiation of ART, adherence to ART, and severe wasting were the most common independent predictors of mortality [1, 3, 17–19]. The evidence suggests that malnutrition has a significant impact on survival of HIV positive children on ART [17, 18], although most of the studies conducted in Ethiopia did not consider baseline nutritional status as an independent predictor. In this study, nutritional status was incorporated as an independent predictor to determine its effect on survival of HIV positive children after initiation of ART. No studies have been conducted in the Amhara Regional State to determine the effect of undernutrition on survival of HIV positive children on ART. Therefore, this study will potentially inform program planners and decision makers at various stages of the HIV/AIDS care and support program. Furthermore, it will serve as a baseline data for further research.

## Methods

### Study area, design and period

An institution-based retrospective cohort study was conducted for the period from the 1st of January, 2012 to the 28th of February, 2017 in Amhara Regional State Referral Hospitals (i.e., Debre Markos Referral Hospital, Felege Hiwot Comprehensive Specialized Hospital, and University of Gondar Comprehensive Specialized Hospital). These three hospitals serve more than 15 million people and care for the largest proportion of HIV clients in the Region. In addition to other services, all three referral hospitals provide chronic HIV care (ART) services. Currently, 1071 children receive ART follow up in these hospitals.

### Population

The source population were all HIV infected children (age < 15 years) whoa recorded initiation on ART in Amhara Regional State Referral Hospitals. The study population were all HIV-infected children who had ART follow up and recorded from the 1st of January, 2012 to the 28th of February, 2017 in any of the three selected hospitals, and whose chart was available during the data collection period. Study participants were taken proportionally from each of the three hospitals based on the total number of children in the ART unit of these hospitals. Children, who received ART for at least for one month between the 1st of January, 2012 to February 28, 2017, were included. However, children with incomplete baseline information were excluded from the study.

### Sample size determination and sampling technique

To calculate the required sample size, severe wasting, severe underweight, and severe stunting were considered as exposure variables. Moreover, severe underweight was considered as an independent variable since it yielded a maximum sample size. The sample size was determined by using a double population proportion formula, and calculated using Open Epi Version 3.To calculate our sample size, the following statistical assumptions were considered:**P1:**percent of exposed (severe underweight) with outcome (12.82%);**P2**:percent among the non-exposed with outcome (4%) estimated from a previous study conducted in Wolaita Health Facility, Southern Nations, Nationalities, and Peoples Region, Ethiopia [18]; **Z α/2**:reflects CI 95%;**Z β**: 80% power; and **ratio** of non-exposed to exposed 1:1. The final sample size was 177 for each group and the total sample size was 354. Finally, after adding 10% for contingency, the final sample size of our study was calculated as 390. The sample was proportionally allocated across each participating hospital, respecting medical records of children based on the previously stated criteria. From the isolated cards in each hospital, simple random sampling technique, computer-generating method was employed to select the study participants. The selected medical charts were followed for five years or until the termination of care.

### Data collection tool and procedure

The data abstraction tool was developed from a standardized ART entry and follow-up form currently used by the ART clinics. To ensure data quality, one-day training was given on the data abstraction tool and data collection process for both data collectors and supervisors. Necessary data were extracted by reviewing patients’ ART cards. The most recent laboratory test results prior to ART initiation were used as a baseline value. Both principal investigators and supervisors closely supervised the entire data collection process. All collected data were examined for completeness and consistency during the data management, storage, and analysis.

### Data processing and analysis

Data were entered into Epi-Data Version 3.1and analysis was done using STATA Version 13 statistical software. The WHO AnthroPlus Version 1.04 and ENA for Smart Software were used to generate the Z score (WAZ, HAZ and WHZ/BAZ) to define nutritional status. The assumption of Cox-proportional hazard regression model was checked using Schoenfeld residual test and variables having *P*-values> 0.1 were considered as fulfilling the assumption criteria. Finally, the outcome of each participant was dichotomized into censored or death. The Kaplan-Meier survival curve was used to estimate survival time after initiation of ART, and log rank tests were used to compare the survival curves. The Bivariate Cox-proportional hazards regression model was fitted for each explanatory variable. Moreover, those variables having *p*-value ≤0.25 in bivariable analysis were fit into the multivariable Cox-proportional hazards regression model. Hazard ratios with 95% confidence interval and *p*-values were used to measure the strength of association and to identify statistically significant predictors. In multivariable analysis, variables having *P*-values< 0.05 were considered as statistically significant predictors of mortality.

### Operational definitions

**Undernutrition** was defined as the child having either of H/Age Z-score < − 2, or W/Age Z-score < − 2 or W/H Z-score < − 2 SD [20, 21].

**Moderate underweight** was defined as children having W/Age Z-score < − 2 SD [20, 21].

**Severe underweight** was defined as children having W/Age Z-score < − 3 SD [20, 21].

**Moderate stunting** was defined as children having H/Age Z-score < − 2 SD [20, 21].

**Severe stunting** was defined as children having H/Age Z-score < − 3 SD [20, 21].

**Moderate wasting** was defined as children having W/H Z-score < − 2 SD [20, 21].

**Severe wasting** was defined as children having W/H Z-score < − 3 SD [20, 21].

**Censored** Individuals on ART but still alive at the end of the study or lost to follow up or individuals transfer out to other health institutions were considered as censored.

**Event** was defined as death of an infant or child after initiation of ART.

### Ethical considerations

Ethical clearance was obtained from an institutional review committee of the School of Nursing, College of Medicine and Health Sciences, University of Gondar. The ethics committee formally waived the need for formal written consent since the study was done through retrospective reviews of patient cards (charts). Permission letters were obtained from each hospital administration of the three participating entities. Since the study was a review of medical records, individual patients were minimally at risk for harm as confidentiality was likely to be achievable. To maintain ‘confidentiality, collected data were coded and locked in a separate room. After entry into the computer, all data were locked by password; as well, names and unique ART numbers were not included in the data collection forms.

## Results

### Socio-demographic characteristics

Among the 390 records included in the final analysis about half (53.9%) were females, and the majority (85.4%) were from urban areas. The mean age of the cohort at the time of ART initiation was 6.9 years (SD ± 0.2 years). The mean age of the caregiver of the children was 32.9 years (SD ± 0.4 year) (Table 1).

**Table 1:** Baseline socio-demographic characteristics of HIV infected children on ART in Amhara Regional State referral hospitals, Ethiopia, January 1st, 2012 – February 28th, 2017

Variables	Frequency (N)	Percentage (%)
Sex		
Male	180	46.1
Female	210	53.9
Age		
< 1 year	11	2.8
1-5 years	116	29.7
5-15 years	263	67.4
Residence		
Urban	333	85.4
Rural	57	14.6
Marital status of caregiver (379)		
Single	44	11.6
Married	191	50.4
Divorced	54	14..3
Widowed	90	23.7
Parental status		
Both alive	223	57.2
Father dead	73	18.7
Mother dead	37	9.5
Both dead	57	14.6
Caregiver of the child		
Parent	324	83.0
Sister/Brother	17	4.4
Uncle/aunt	9	2.3
Grandparent	25	6.4
Others	15	3.9

### Clinical characteristics of study participants

Clinically more than half (58.8%) of the children started ART when classified as ‘mild’ according to the WHO HIV clinical disease stage (I&II). About three quarters (75.8%) of the children had achieved appropriate developmental milestones prior to ART initiation. Prior to ART initiation, 53.3% of the children had opportunistic infections. More than two thirds (70.0%) of the children had CD4 counts below the threshold for severe immunodeficiency. About 30.3% of the children had a history of regimen change throughout the entire follow up. From those who had a history of regimen change, toxicity or drug side effect (44.8%) was the most common reason followed by drug out of stock (24.6%) and clinical failure (9.8%). In addition, among those experiencing drug side effects (17.8%) during the follow-up period, anemia (30.8%) was the most common complaint followed by nausea (20.8%) and rash (12.3%) (Table 2).

**Table 2:** Baseline clinical and immunological profile of HIV positive children on ART in Amhara Regional State referral hospitals, Ethiopia from the 1st of January, 2012 – the 28th February, 2017

Variables	Frequency (N)	Percentage (%)
OI at baseline		
Yes	182	53.3
No	208	46.7
Functional status (age ≥5 year) (*N* = 262)		
Working	150	57.3
Ambulatory	105	40.1
Bedridden	7	2.7
Developmental History (age < 5 years) (*N* = 128)		
Appropriate	97	75.8
Delayed	24	17.8
Regressive	7	5.5
WHO clinical stage		
Stage I and II	229	58.8
Stage III and IV	161	41.2
Past TB test before ART		
Not determined	310	79.5
Negative	37	9.5
Positive	43	11.0
Past TB treatment		
No treatment	346	88.7
2SRHZ/4RH	30	7.7
2HRZES/4HRE	3	0.8
HRZE/4RH	11	2.8
CD4 count or percent		
Below the threshold	273	70.0
Above the threshold	117	30.0
Hemoglobin level		
< 10 g/dl	47	12.0
≥10 g/dl	343	88.0
ART eligibility criteria		
Immunologic/ CD4	107	27.9
WHO clinical stage	47	12.0
Both clinical and immunologic	152	39.0
Without criteria	84	21.5
Type of Regimen during follow up		
4a = d4t-3TC-NVP	48	12.3
4b = d4t-3TC-EFV	22	5.6
4c = AZT-3TC-NVP	149	38.2
4d = AZT-3TC-EFV	78	20.0
1e = TDF-3TC-EFV	35	9.0
1 h = ABC-3TC-NVP	10	2.6
1 g = ABC-3TC-EFV	13	3.3
2nd line	9	2.3
Others	26	6.7
OI during follow-up		
Yes	107	27.4
No	283	72.6
Cotrimoxazole preventive therapy		
Yes	233	59.7
No	157	40.3
Regimen change during follow-up		
Yes	118	30.3
No	272	69.7
Regimen stopped during follow-up		
Yes	18	4.6
No	372	95.4
ART adherence in the 1st 3 months		
Good /fair	495	92.2
Poor	42	7.8

### Nutritional status of the study participants

At baseline, about one-fifth (20.3%) of the study participants were moderately underweight, 12.0% were moderately stunted, and 12.6% were moderately wasted. In addition, the proportions of presenting child malnutrition cases were classified as severe stunting (19.7%), severe wasting (13.9%), and severe underweight (15.1%) (Table 3).

**Table 3:** Baseline nutritional status of HIV positive children on ART in Amhara Regional State referral hospitals, Ethiopia from the 1st of January, 2012 – the 28th February, 2017

Variables	Frequency (N)	Percent (%)
Underweight		
Normal	252	64.6
Moderate (WAZ < − 2)	79	20.3
Severe (WAZ < −3)	59	15.1
Stunting		
Normal	266	68.2
Moderate (HAZ < −2)	47	12.0
Severe (HAZ < − 3)	77	19.7
Wasting		
Normal	287	73.6
Moderate (WHZ or BAZ < − 2)	49	12.6
Severe (WHZ or BAZ < −3)	54	13.9

### Incidence of mortality

Within a median follow-up period of 24.65 months with IQR (12- 39 months), 9.7% of the study participants died, 5.4% were lost to follow up, 14.4% were transferred out, leaving the remaining 70.5% alive. The overall mortality rate of the entire follow-up was 4.4 per 100 child years (95% CI: 3.2, 6.0). Regarding the time of death, 50%, 63%, and 68% of the deaths occurred within the first six, twelve, and eighteen months of ART initiation, respectively. The mean survival time of the entire follow-up was 56.4 months (95% CI: 53.7, 57.5). The cumulative probabilities of survival at 3, 6, 12, 24, and 60 months after initiation of ART were 0.966, 0.950, 0.935, 0.831, and 0.831, respectively. The cohort contributed a total of 10,376 child-months of follow-up.

### Survival function of different predictor variables

**Fig. 1:**
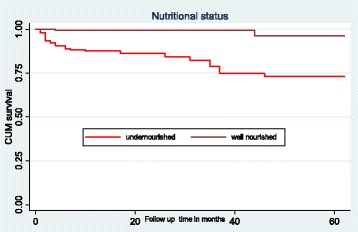
The Kaplan-Meier survival curves to compare survival time of HIV positive children on ART with different categories of nutritional status in Amhara Regional State referral hospitals, Northwest Ethiopia from January 1st, 2012- February 28th, 2017

To test the equality of survival curves for different categorical predictor variables the Cochran-Mantel Haenszel log rank test was used. The results showed that there was a significant difference in the survival function of different categorical variables; specifically, nutritional status (under or normal), hemoglobin (< 10 g/dl and ≥10 g/dl), CD4 count or percent (below and above the threshold), and WHO HIV clinical disease staging (i.e., mild to advanced).The mean survival time for children who were undernourished at the time of ART initiation was 50.6 months (SD±1.7), but 61.1 months (SD ± 0.6) for those who were well nourished. This difference was statistically significant with a *P*-value < 0.001 (Fig. 1).

**Fig. 2:**
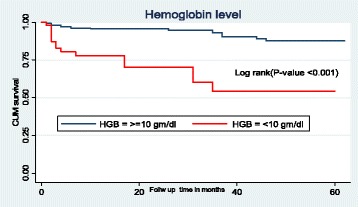
The Kaplan-Meier survival curves to compare survival time of HIV positive children on ART with different categories of hemoglobin level in Amhara Regional State referral hospitals, Northwest Ethiopia from January 1st, 2012- February 28th, 2017

In this study, children who had low hemoglobin levels (< 10 g/dl) had a lower survival time as compared to those with high hemoglobin level (≥10 g/dl). The mean survival time of children having low hemoglobin level was 39.6 months (SD±4.1) and the mean survival time of children having hemoglobin level ≥10 g/dl was 57.5 months (SD±0.9). This difference was statically significant with *P*-value < 0.001 (Fig. 2).

**Fig. 3:**
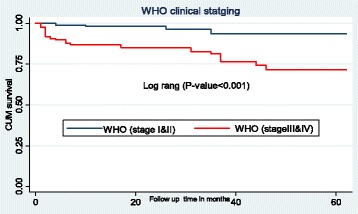
The Kaplan-Meier survival curves to compare survival time of HIV positive children on ART with different categories of WHO clinical staging in Amhara Regional State referral hospitals, Northwest Ethiopia from January 1st- February 28th, 2017

The mean survival time for children classified as WHOHIV clinical stages I or II at the time of ART initiation was 59.5 months (SD±0.9) as compared to those assessed as WHO HIV clinical stages III and IV with a mean survival time of 50.3 months (SD±1.9). This difference is statistically significant with *P*-value < 0.001 (Fig. 3).

**Fig. 4:**
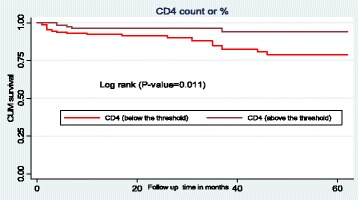
The Kaplan-Meier survival curves to compare survival time of HIV positive children on ART with different categories of CD4 count or percent in Amhara Regional State referral hospitals, Northwest Ethiopia from January 1st- February 28th, 2017

The mean survival time for initiates withCD4 count or percent below the threshold was 53.9 months (SD ±1.3), which was lower than the mean survival time of individuals who had CD4 count or percent above the threshold with a mean survival time of 59.3 months (SD ±1. 2). This difference was statistically significant with P-value = 0.011 (Fig. 4).

### Bivariable and multivariable cox-regression analysis

In the bivariable analysis, being male, aged 5-15 years, living in rural areas, hemoglobin level < 10 g/dl, presence of OI, CD4 cell count or percentage below the threshold, categorized as WHO HIV clinical stages III&IV, underweight (moderate and severe), stunted (moderate and severe) and wasted (moderate and severe) were found to be significant predictors of mortality. In the multivariable Cox-regression analysis, only five variables were found to be predictors of mortality. The result of multivariable analysis revealed that children with advanced WHO HIV clinical stages (III and IV) were 2.6 times higher risk of death as compared to those with WHO HIV clinical stage (I and II) (AHR: 2.6, 95% CI: 1.1, 6.6). In this study, CD4 count was found to be another predictor of mortality. Children exhibiting CD4 cell count or percentages below the threshold were 5.2 times at higher risk of death as compared to cohort members below thresholds (AHR: 5.2, 95% CI: 1.9, 14.1). Moreover, initiates with hemoglobin levels less than 10 g/dl were at 3.2 times higher risk of death than those with hemoglobin levels greater than or equal to 10 g/dl (AHR: 3.2, 95% CI: 1.4, 7.4). Case presence of severe stunting at the beginning of ART showed 3.9 times higher risk of death than non-stunting cases (AHR: 3.9, 95% CI: 1.7, 9.4). Furthermore, children who were severely wasted were at 3.0 times higher risk of death as compared to those who were not wasted (AHR: 3.0, 95% CI: 1.3, 6.9) (Table 4).

**Table 4:** The bivariable and multivariable Cox regression analysis of predictors of mortality among HIV positive children on ART in Amhara Regional State referral hospitals, Ethiopia from January 1st, 2012 - February 28th, 2017

Variables	Survival status Dead Censored		CHR (95%CI)	AHR (95%CI)
Sex				
Male	23	154	2.5 (1.3, 5.0)	1.3 (0.6, 3.0)
Female	15	198	1	1
Age				
< 1yer	3	8	4.9 (1.4, 17.0)	2.5 (0.6, 9.9)
1-5 years	17	99	2.2(1.1, 4.3)	1.7(0.7, 3.7)
5-15 years	18	245	1	1
Residence				
Urban	35	298	1	1
Rural	3	54	0.6(0.2, 2.0)	0.5(0.1, 1.7)
OI at baseline				
Yes	30	152	4.3 (2.0,9.0)	1.9 (0.8, 4.5)
No	8	200	1	1
WHO clinical staging				
Stage I and II	8	221	1	1
Stage III and IV	30	131	5.6 (2.6, 12.3)	2.6 (1.1, 6.6)^**^
CD4 count or percent				
Below the threshold	33	2410	3.2 (1.2, 8.2)	5.2 (1.9, 14.1)^**^
Above the threshold	5	112	1	1
Hemoglobin level				
< 10 g/dl	15	32	6.2 (3.2, 11.9)	3.2 (1.4, 7.4)^**^
≥ 10 g/dl	23	320	1	1
Underweight				
Normal	10	242	1	
Moderate (WAZ < − 2	13	66	4.2 (1.8, 9.6)	2.4 (0.9,6.4)
Severely (WAZ < −3)	15	44	6.8 (3.01, 15.2)	1.3 (0.5, 3.6)
Stunting				
Normal	12	254	1	1
Moderate (HAZ < −2)	8	39	3.9 (1.6, 9.7)	2.3 (0.9, 6.29)
Severe (HAZ < −3)	18	59	6.1 (2.9, 12.9)	3.9 (1.7, 9.4)^**^
Wasting				
Normal	18	269	1	1
Moderate (WHZ or BAZ < −2)	5	44	1.5 (0.5, 4.0)	1.2 (0.4, 3.6)
Severe (WHZ or BAZ < −3)	15	39	4.5 (2.2,9.0)	3.0 (1.3, 6.9)^**^

**Significant predictors in the multivariable analysis

## Discussion

In this retrospective cohort study, the incidence and predictors of mortality were determined among HIV positive children on ART. At the end of follow-up, about 9.7% patients were deceased and 5.4% patients were lost to follow-up. The overall mortality rate of this study was 4.4 per 100 child-years(95% CI: 3.2, 6.0) which is consistent with findings in Congo (3.4 deaths per 100 child-years) [22] and at Bahir Dar Referral Hospital, Northwest Ethiopia (four deaths per 100 child-years) [1]. However, the mortality rate reported in this study is higher than studies reported from Mekelle Hospital, Northern Ethiopia (1.4 deaths per 100 child-years) [23], Zimbabwe (2.9 deaths per 100 child-years) [24], Wolaita Zone health facilities, Southern Ethiopia (2.1 deaths per 100 child-years) [18], and the Asia Pacific region (1.9 deaths per 100 child-years) [25].Conversely, the mortality rate found in this study is much lower than a Kenyan study where the findings reflected 8.4 deaths per 100 child-years [26].

Explanations for variation in the incidence of mortality rate might be due to the difference in sample size, study settings, study period, and/or characteristics of study participants. The higher mortality in the current study may relate to the fact that more than two-thirds (70.0%) of participants had CD4 counts or percentages below the threshold for severe immunodeficiency at the time of ART initiation. Children with CD4 counts or percentages below the threshold are at higher risk of developing opportunistic infections, which escalates the possibility of death during the early phase of treatment prior to responding to HAART drugs. Another possible explanation might relate to this study being conducted in referral hospitals, which provide tertiary level services for patients from health centers or general hospitals that are often experiencing advanced disease stage and complexity of management for ART initiation. These factors individually and collectively could increase the incidence of mortality.

The aim of this study was to determine the effect of nutritional status of undernourished children was much lower than well-nourished children (61.1 months with SD ±0.6).Supportive findings was reported from a study conducted in Singapore [27]. According to this study, malnutrition was significantly associated with reduced survival of patients taking ART. It is known that undernourished children are more prone to develop opportunistic infections than well-nourishedchildren. The presence of opportunistic infections during ART initiation increase piles burden, which results in drug-drug interactions and potentiates early death after ART initiation either due to drug side effect or due to opportunistic infections.

Concerning the interval from ART initiation to death, 63% of the deaths occurred within the first 12 months. In this study, early mortality (death ≤12 months of ART initiation) was higher than late mortality (death ≥ 12 months after ART initiation) with an incidence rate of 7.2 per 100 child-years and 2.4 per 100 child-years respectively. This finding is consistent with a study conducted in Mekelle Hospital, Northern Ethiopia, which showed that early mortality (death < 18 months after ART initiation) was higher than late mortality (death ≥18 months after ART initiation [23]. This finding is also in line with other studies conducted in other sub-Saharan Africa countries which showed that most of the deaths occurred in the first 6 months following ART initiation [28, 29].This high early mortality after ART initiation in our study area might be attributed to the proportion (70%) of the patients having CD4 counts or percentages below the threshold for severe immunodeficiency at the time of ART initiation. Patients with severe immunodeficiency are greatly affected by various types of opportunistic infections and which could compromise proper treatment outcomes and may accounts for our findings.

The present study explored predictors of mortality among study participants. In this study, low hemoglobin level was found to be a predictor of mortality among children living with HIV on ART. Children who had low hemoglobin levels at the time of ART initiation have a higher risk of death as compared to those who had hemoglobin levels≥10 g/dl. Previous studies from Ethiopia reported that low hemoglobin level was a strong predictor of mortality [1, 17, 23, 30], and studies from other sub-Saharan African countries also demonstrated that low hemoglobin was a predictor of mortality [26, 29, 31]. In this study, nearly 58.2% of our study participants were taking the drug of 4c (AZT-3TC-EFV) or 4d (AZT-3TC-NVP) during the follow-up time. Zidovudine (AZT) is one of the most common causes of anemia (megaloblastic anemia) among patients living with HIV. Cotrimoxazole preventive therapy (CPT) might be another cause of anemia among children living with HIV, in which more than half (63.2%) of participants have been taking CPT.

Children, who had CD4 counts or percents below the threshold, yet another strong predictor of mortality, showed a higher risk of death than their counterparts. This finding is consistent with other previous studies conducted in Ethiopia [1, 17, 23], India [32], Congo [33], Tanzania [31], Bangladesh [34] and Malaysia [35]which all indicate that low CD4 count was an independent predictor of mortality. Children with severe immunodeficiency are at higher risk of developing serious and life-threatening opportunistic infections, such as CNS toxoplasmosis and cryptococcal meningitis.

An advanced WHO HIV clinical staging (III and IV) was also an independent predictor of mortality. Children with advanced WHO HIV clinical stage (III and IV) at the time of ART initiation have a higher risk of death as compared to their counterparts with mild status (i.e., WHOHIV clinical disease stage (I and II)). Similar results were reported from previous studies conducted in Ethiopia [23, 30, 36], Kenya [26], Malawi [37] and Zimbabwe [24]which all indicated that advanced WHO HIV clinical disease stages was a predictorof mortality. For those living with HIV, as WHO HIV clinical staging becomes more advanced, the risk of developing and recurrence of opportunistic infection also simultaneously increased, which might be associated with the cause of death.

In this study, severe stunting was another predictor of mortality among HIV positive children on ART. Children who were severely stunted prior to ART initiation have a higher risk of death as compared to those who were not stunted. Reasonably, malnutrition is a common complication of HIV infection. Severe stunting is associated with a weakened immune system and complicates the treatment of diseases by affecting intestinal absorption of drugs and the ability to absorb various nutrients [31].

Furthermore, being severely wasted prior to ART initiation was a strong predictor of mortality. Those who were severely wasted prior to ART initiation have a higher risk of death as compared to those who were not wasted. Comparable reports were also noted in previous in Ethiopia [17, 18]. A study from Kenya revealed that (WHZ < − 2) increased the risk of death among HIV positive children receiving HAART [26]. Similarly, a study from Tanzania reported (WHZ and BAZ of ≤ -2) significantly increased the risk of mortality in the first 90 days of ART initiation [31]. Generally, many factors place HIV patients at higher risk of developing undernutrition than the general population. Factors such as reductions of food intake, poor absorption of nutrients that may be the result of recurrent or chronic diarrhea, HIV-caused intestinal cell damage, increased energy needs because of virus replication and opportunistic infections (OIs), have been previously recognized [12, 38].

### Study limitations and strengths

This study experienced inconsistencies in ascertaining causes of death, especially for those who died at home. Loss to follow-up might also have included those died without being reported. Furthermore, important predictors of mortality, like viral load and micronutrient deficiency, were not considered. Despite the above limitations, the study was conducted over a significant period, which increases the period of observation, thereby increasing the number of events.

## Conclusion

The findings of this study indicated that there was a high rate of mortality among HIV positive children on ART. In this study, nutritional status was found to have a significant effect on the survival of HIV positive children on ART. Anemia, baseline CD4 count or percent below the threshold, advanced WHO HIV clinical disease staging (III and IV), severe stunting, and severe wasting were found to be independent predictors of mortality among HIV children on ART. Therefore, studying the impacts of maximizing nutritional supplements and other nutritional interventions are needed to be encouraged with special emphasis HIV positive children.

## Abbreviations

AIDS: Acquired Immune Deficiency Syndrome
HAZ: Height for Age Z score
Hgb: Hemoglobin
HIV: Human Immunodeficiency Virus
WAZ: Weight for Age Z score
WHO: World Health Organization.
WHZ: Weight for Height Z score
